# Antinociceptive Effect of Cinnamaldehyde in Male Mice: Investigation of the Mechanisms of Action Through In Silico and In Vivo Approaches

**DOI:** 10.1002/cbdv.202503228

**Published:** 2026-04-27

**Authors:** Renaly I. de A. Rêgo, Hugo F. O. Pires, Arthur L. Dias, Maria Caroline R. B. Remigio, Humberto H. N. de Andrade, Pablo R. da Silva, Natália F. de Sousa, Luciana Scotti, Marcus T. Scotti, Mirian G. S. S. Salvadori, Ricardo D. de Castro

**Affiliations:** ^1^ Postgraduate Program in Natural and Synthetic Bioactive Products Center of Health Science Federal University of Paraíba (UFPB) João Pessoa Paraíba Brazil; ^2^ Institute of Research in Drugs and Medicines Federal University of Paraíba (UFPB) João Pessoa Paraíba Brazil

**Keywords:** cinnamaldehyde, natural products, neurochemistry, nociception, pain, phenylpropanoid

## Abstract

Cinnamaldehyde (CA), a major component of Cinnamomum spp. essential oils, has recognized bioactivity, including possible analgesic effects. However, its acute antinociceptive mechanisms remain unclear. This study assessed the influence of CA (15, 30, and 60 mg/kg, p.o.) on locomotor and exploratory behaviors via rotarod and open field tests in male Swiss mice. Antinociceptive activity was evaluated using chemical and thermal nociception models. Mechanistic investigations were performed in the opioid and adrenergic systems, as well as in silico molecular docking in nociceptive targets. A significance level of *p* = 0.01 was adopted. CA significantly reduced nociceptive behaviors in all models without impairing motor coordination. In the formalin test, it inhibited neurogenic and inflammatory phases. In glutamate and capsaicin tests, CA markedly reduced nociceptive responses. In the hot plate test, it increased latency at 30 mg/kg. Naloxone reversed the antinociceptive effect of CA in the formalin test, supporting the hypothesis of opioid receptor involvement, while yohimbine partially blocked the response, suggesting α_2_‐adrenergic contribution. Docking simulations showed favorable interactions of CA with κ‐ and δ‐opioid receptors, NMDA, AMPA, and TRPV1, supporting a multimodal mechanism. CA displays acute antinociceptive activity, and the findings suggest the involvement of central pathways, including opioid and adrenergic systems.

## 1 Introduction

Pain is a multisensory experience involving both physical and emotional aspects, associated with actual or potential tissue damage [1]. Classified as one of the most significant causes of human suffering and the leading reason for clinical consultations worldwide, pain represents a major public health issue that affects quality of life, sleep, work capacity, social interactions, and increases healthcare use, costs, and mortality rates. Epidemiological studies have shown that chronic pain can contribute to psychiatric disorders such as depression, anxiety, and schizophrenia, mainly due to its complexity [2]. Pain is considered a multifaceted experience that encompasses not only the transduction of nociceptive stimuli but also the cognitive and emotional processing by the brain, which governs various behavioral responses [3].

Despite the wide range of substances available and progress in pain management therapies, the search for new therapeutic strategies that are both effective and associated with minimal adverse effects remains urgent, especially for intense pain conditions, in which morphine is still the most effective pharmacological option, although it is associated with undesirable effects such as respiratory depression, sedation, euphoria/reward, nausea, urinary retention, biliary spasm, and constipation [4].

Natural products are a rich source of bioactive compounds with therapeutic potential due to their chemical diversity. Historically, they have greatly advanced pharmacotherapy, remaining a primary source of new drugs. Between 1981 and 2019, 1,881 new drugs were FDA‐approved, with 49.2% derived from natural products [5]. Medicinal plants produce compounds that modulate organ and system functions, making them valuable for discovering new therapies [6].

Cinnamaldehyde (CA), a phenylpropanoid found in the essential oil of Cinnamomum species, is widely used in foods and recognized as safe by the FDA. Studies have shown that CA has neuroprotective, anxiolytic, and antioxidant effects [7]. Intra‐amygdala administration of CA has been shown to reduce pain perception in sleep‐deprived rats, supporting the hypothesis that this compound possesses antinociceptive activity [8]. Given that cinnamaldehyde exhibits affinity for transient receptor potential (TRP) channels, particularly the ankyrin subtype 1 (TRPA1), which is abundantly expressed in the gastrointestinal tract, it is plausible that its interaction with these receptors also contributes to the modulation of pathophysiological processes related to nociception [9]. In line with these findings, our group previously demonstrated that cinnamyl alcohol, a structural analog of CA, significantly reduced paw‐licking behavior in both the neurogenic and inflammatory phases of the formalin test, supporting its antinociceptive activity [10]. These findings further strengthen the hypothesis that CA exerts antinociceptive effects through receptor‐mediated modulation of nociceptive signaling pathways.

Thus, the study aimed to evaluate the antinociceptive potential of cinnamaldehyde in mice and contribute to the elucidation of its mechanism of action through experimental protocols and in silico molecular docking simulations.

## 2 Results

### 2.1 Molecular Docking Analysis

Molecular docking was performed using the Moldock Score as the scoring function, where more negative values indicate stronger predicted binding affinity [11]. Proteins in which cinnamaldehyde showed lower binding energy values or values comparable to the co‐crystallized PDB ligand in at least one scoring function were considered potential targets and possible mechanisms of action [12]. Cinnamaldehyde exhibited affinity values (Table S2) close to the control compounds and PDB ligands for cyclooxygenase‐1 (PDB: 6Y3C), NMDA receptor (PDB: 4NF5), AMPA receptor (PDB: 5ZG0), α_2_‐adrenergic receptor (PDB: 5FJV), and TRPV1 channel (PDB: 5IS0). It is important to emphasize that the results obtained through molecular docking simulations consist of theoretical findings, which provide exclusively predictions and putative binding modes, and their confirmation through experimental data is essential.

Before performing the molecular docking simulations, the targets under study were validated through redocking between the ligands and the co‐crystallized proteins. During the redocking analysis, it was observed that the Root Mean Square Deviation (RMSD) value was below 2.0 Å (Table S2), meaning that the generated pose correctly positioned the ligand at the active site. This indicates that the program provided values considered satisfactory for docking validation.

For the opioid receptors K (PDB: 4DJH), μ (PDB: 6DDF), and σ (PDB: 6PT3), as well as for the metabotropic glutamate receptor (PDB: 4OO9) and the COX‐2 enzyme (PDB: 6COX), no binding energy values lower or close to those of positive controls and/or PDB ligands were observed. This difference may be related to molecular weight, since the Moldock Score algorithm takes into account the number of heavy atoms in the chemical structure. Cinnamaldehyde has a molecular weight of 132.16 g/mol, which is considered low compared to the controls, such as deltorphin II (782.9 g/mol). To evaluate the influence of this variable on affinity score calculations, normalization of the score as a function of molecular weight was applied, known as Docking Score Correction (DSC), calculated as the ratio between the affinity score value and the molecular weight of the compound [13]. Table S3 presents the DSC values for cinnamaldehyde in the analyzed targets.

According to Table S3, molecular weight influenced the binding energy values. For opioid receptors, as well as for the metabotropic glutamate receptor and the COX‐2 enzyme, normalization of affinity scores indicated that cinnamaldehyde presented the highest theoretical affinity among the compounds analyzed.

**FIGURE 1:**
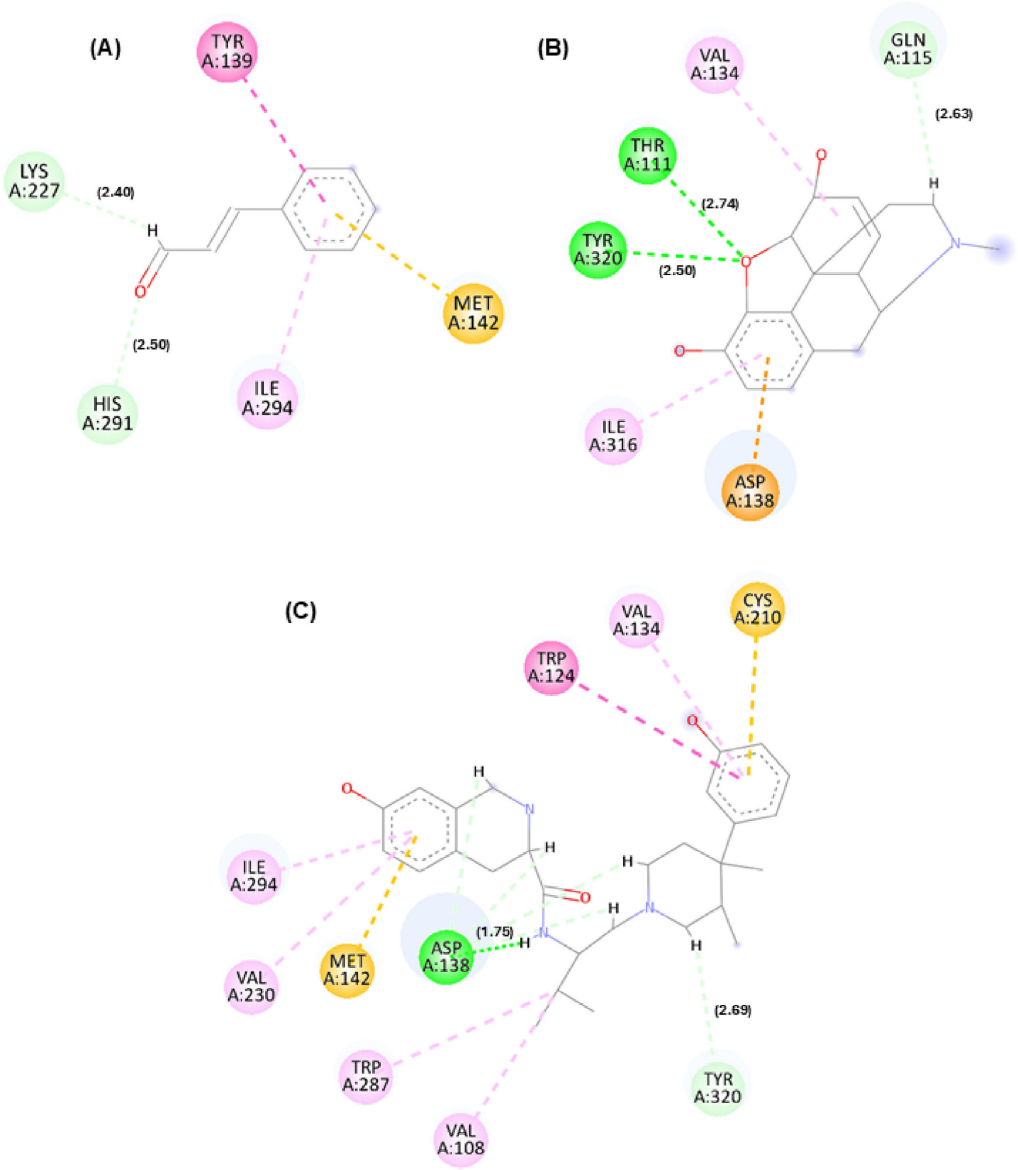
Molecular interaction of the cinnamaldehyde compound with K opioid receptors. Legend: 2D interactions for the compounds cinnamaldehyde (A), morphine (B), and tetrahydroisoquinoline derivative PDB ligand (C) with the μ opioid receptor target (PDB: 6DDF). Interactions: dark pink (Pi–Pi T‐shaped), (light pink) akyl and Pi‐alkyl, yellow (Pi‐sulfur), light green (carbon hydrogen bond), orange (Pi‐anion), dark green (conventional hydrogen bond). Residues: Tyr (tyrosine), Lys (lysine), His (histidine), Ile (isoleucine), Met (methionine), Val (valine), Thr (threonine), Asp (aspartic acid), Cys (cysteine), and Trp (tryptophan). Pipeline pilot BIOVIA was used to generate the image.

**FIGURE 2:**
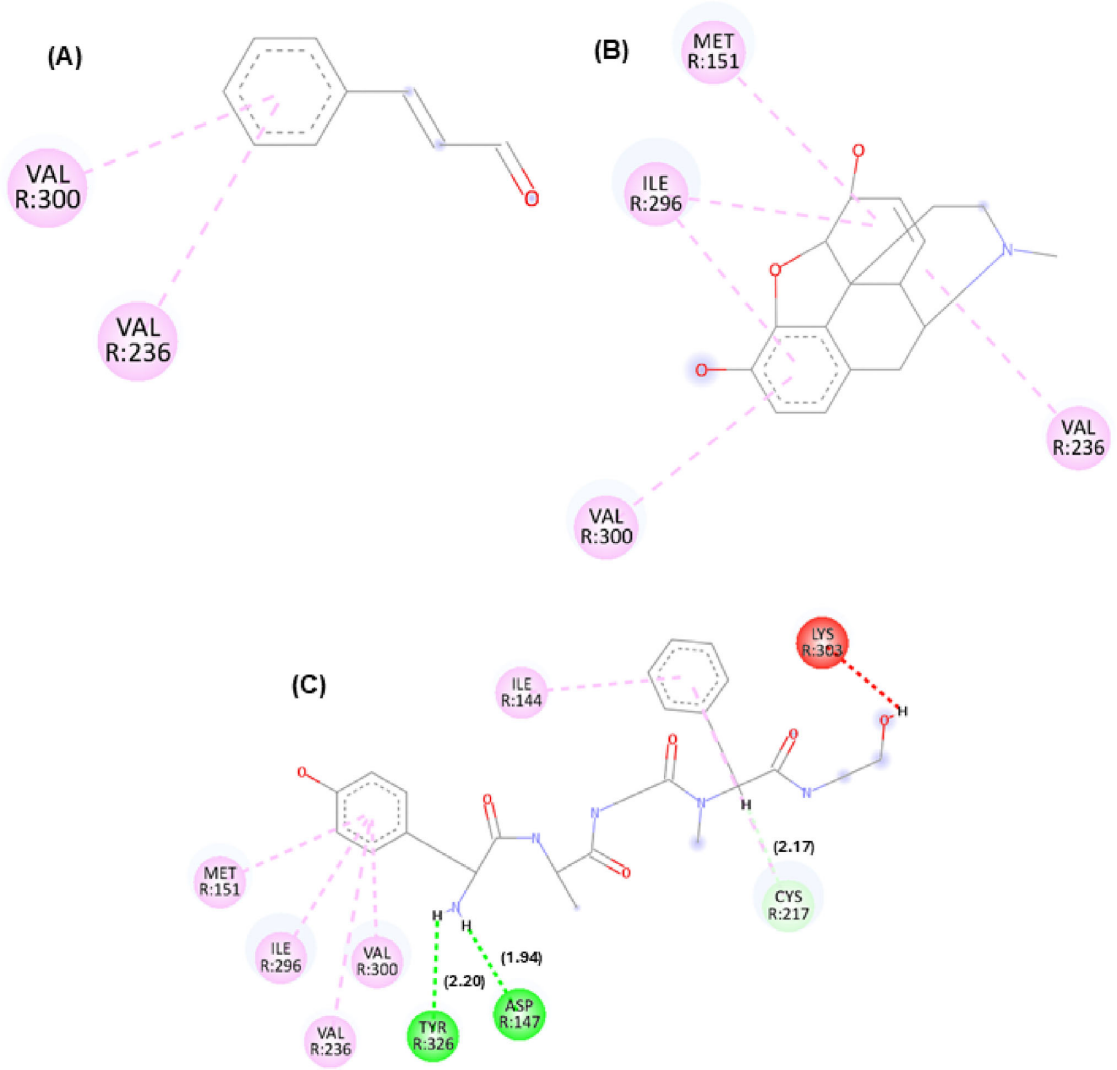
Molecular interaction of the cinnamaldehyde compound with μ opioid receptors. Legend: 2D interactions for the compounds cinnamaldehyde (A), morphine (B), and PDB Ligand PDR_002308 (C) with μ (Mu) opioid receptor target (PDB: 6DDF). Interactions: light pink (akyl and Pi‐alkyl), light green (carbon hydrogen bond), dark green (conventional hydrogen bond), and red (unfavorable interaction). Residues: Val (valine), Met (methionine), Ile (isoleucine), Lys (lysine), Cys (cysteine), Asp (aspartic acid), and Tyr (tyrosine). Pipeline pilot BIOVIA was used to generate the image.

**FIGURE 3:**
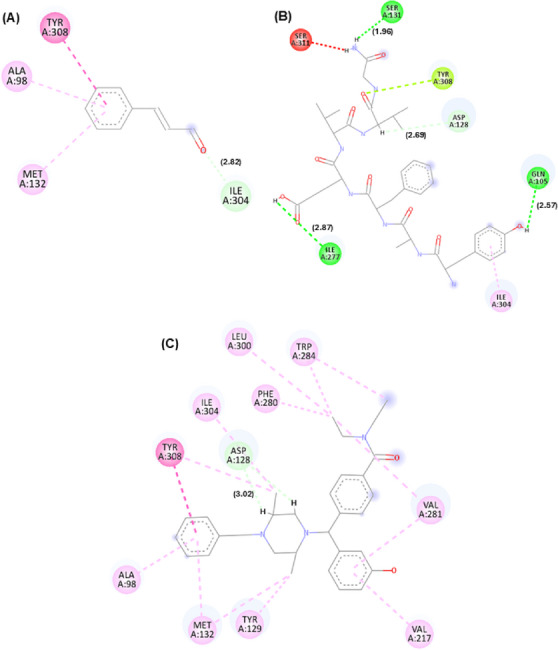
Molecular interaction of the cinnamaldehyde compound with δ opioid receptors. Legend: 2D interactions for the compounds cinnamaldehyde (A), morphine (B), and PDB Ligand PDR_002308 (C) with the μ (Mu) opioid receptor target (PDB: 6DDF). Interactions: Light pink (akyl and Pi‐alkyl), light green (carbon hydrogen bond), dark green (conventional hydrogen bond), and red (unfavorable interaction). Residues: Val (valine), Met (methionine), Ile (isoleucine), Lys (lysine), Cys (cysteine), Asp (aspartic acid), and Tyr (tyrosine). Pipeline pilot BIOVIA was used to generate the image.

In addition to affinity scores, interaction maps between cinnamaldehyde and these targets were generated. The best docking pose orientations are shown in Figures 1, 2, 3, while additional poses are provided in the supplementary material.

Although cinnamaldehyde exhibited the highest normalized binding affinity for COX‐2 (Table S3), this finding is based exclusively on in silico docking analyses. No in vivo or biochemical assays evaluating cyclooxygenase activity or inflammatory mediators were performed in the present study. Therefore, the predicted interaction with COX‐2 should be interpreted as a hypothesis‐generating result that expands the exploration of potential mechanistic pathways, rather than direct evidence of cyclooxygenase involvement in the observed antinociceptive effects.

According to Figures 1, 2, 3, it is possible to observe that the theoretical molecular interaction maps related to opioid receptors indicate a greater contribution of hydrophobic interactions, which is consistent with previously described characteristics of this class of receptors that correspond to the presence of binding sites characterized by hydrophobic cavities that may favor interactions with lipophilic ligands such as morphine and its derivatives [14]. For the κ receptor, hydrophobic interactions of the π–π T‐shaped type were predicted through residue Tyr139 (dashed line in dark pink) and π–alkyl (dashed line in light pink) through residue Ile294, as well as a steric interaction (dashed line in orange) through residue Met142. In addition, hydrogen bond‐type interactions were identified (represented by green dashed lines), involving residues Lys227 and His291. These residues are part of the predicted binding cavity corresponding to the binding site of both the antagonist and morphine in the kappa receptor. Also noteworthy is the residue Ile294, previously described as relevant for the structural organization of this site in the receptor [15].

For the μ receptor, it was observed that the cinnamaldehyde compound was predicted to engage primarily with the benzene group, which was associated with the occurrence of hydrophobic interactions (dashed pink line) through residues Val300 and Val236. It is also important to note that the residues presented were observed in the compound morphine and in the PDB ligand, suggesting a potential overlap in predicted binding regions, demonstrating that the three compounds may share overlapping sites. For the δ receptor, similar to what was identified in the κ receptor, the interaction of the ketone groups was predicted through the oxygen atom (O), establishing carbon–hydrogen bonds (represented by light green lines) with the residue Ile304. Furthermore, hydrophobic interactions of the alkyl and π–alkyl type (light pink line) were detected through residues Ala98 and Met132, as well as π–π stacked type interactions (dark pink line) through residue Tyr308, which has been described as relevant for ligand recognition within the receptor [16].

### 2.2 Behavioral Screening

Pharmacological behavioral screening evaluates behavioral parameters proposed by Almeida (1999) [17] for investigating the psychopharmacological activity of new molecules, in order to outline the profile of the effects of cinnamaldehyde on the central and autonomic nervous system [18]. The behavioral changes presented by mice treated with CA in relation to the control are shown in Table S4. At a dose of 15 mg/kg, after 30 min of treatment, the animals presented analgesia, which persisted until 60 min. At 120, 180, and 240 min, no effects resulting from the treatment were observed.

The animals that received the dose of 30 mg/kg, after 30 min of treatment, presented analgesia, which persisted until 120 min. At 180 and 240 min, no effects resulting from the treatment were observed. At a dose of 60 mg/kg, analgesia was observed at 30 min, which persisted until 180 min. No other effects were observed. The decrease in algesia was assessed by increasing the latency in the animal's response to pressure on the lower third of the tail. This effect was observed in all doses analyzed, indicating that tests to evaluate the antinociceptive activity of cinnamaldehyde would provide promising results.

Based on these data indicating an analgesic effect, the next step in the study was to perform antinociceptive tests. Furthermore, none of the doses tested were capable of causing animal death.

### 2.3 Open Field Test

Cinnamaldehyde significantly reduced ambulation in the open field test at all evaluated doses when compared to the control group: 15 mg/kg (mean difference = 23.50, *p* = 0.0117), 30 mg/kg (mean difference = 29.33, *p* = 0.0013), and 60 mg/kg (mean difference = 50.67, *p* < 0.0001). The most pronounced effect was observed at 60 mg/kg, corresponding to a 67.19% reduction in locomotor activity relative to the control.

**FIGURE 4:**
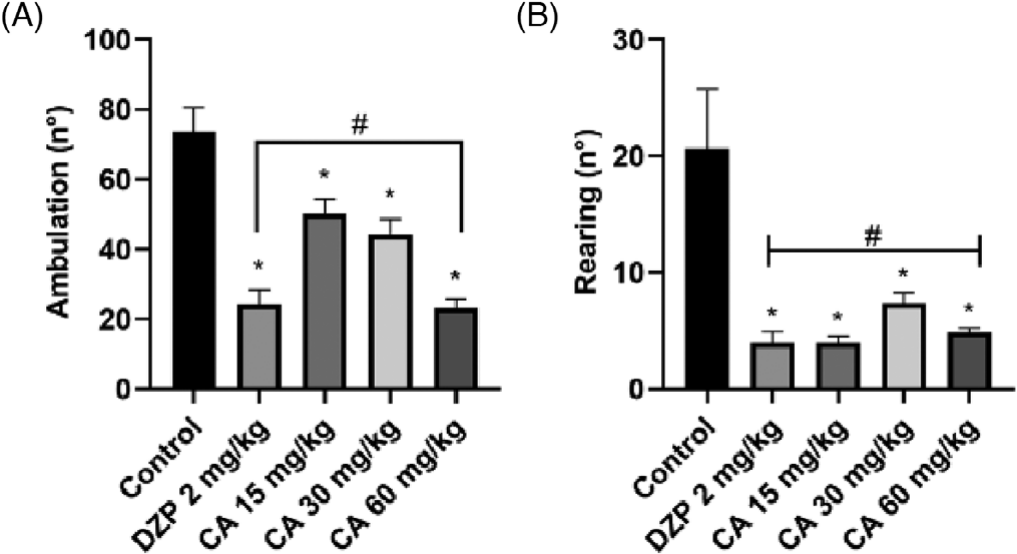
Effect of treatment with cinnamic aldehyde (15, 30, and 60 mg/kg; p.o) and DZP (2 mg/kg; i.p) on the parameters of ambulation (a) and rearing (b) in the open field test. Legend: Each column represents mean ± standard deviation (*n* = 6), being compared by analysis of variance (ANOVA), followed by Tukey's test. *p <0.01 vs control. Locomotor activity‐rotarod test.

A significant reduction in vertical exploratory activity (rearing) was also observed following treatment with cinnamaldehyde. All tested doses led to statistically significant decreases compared to the control group: 15 mg/kg (*p* < 0.0001), 30 mg/kg (*p* < 0.0001), and 60 mg/kg (*p* < 0.0001). Notably, there was no significant difference in rearing between animals treated with CA at 60 mg/kg and those receiving diazepam (*p* = 0.9846). These findings are presented in Figure 4.

### 2.4 Locomotor Activity‐Rotarod Test

**FIGURE 5:**
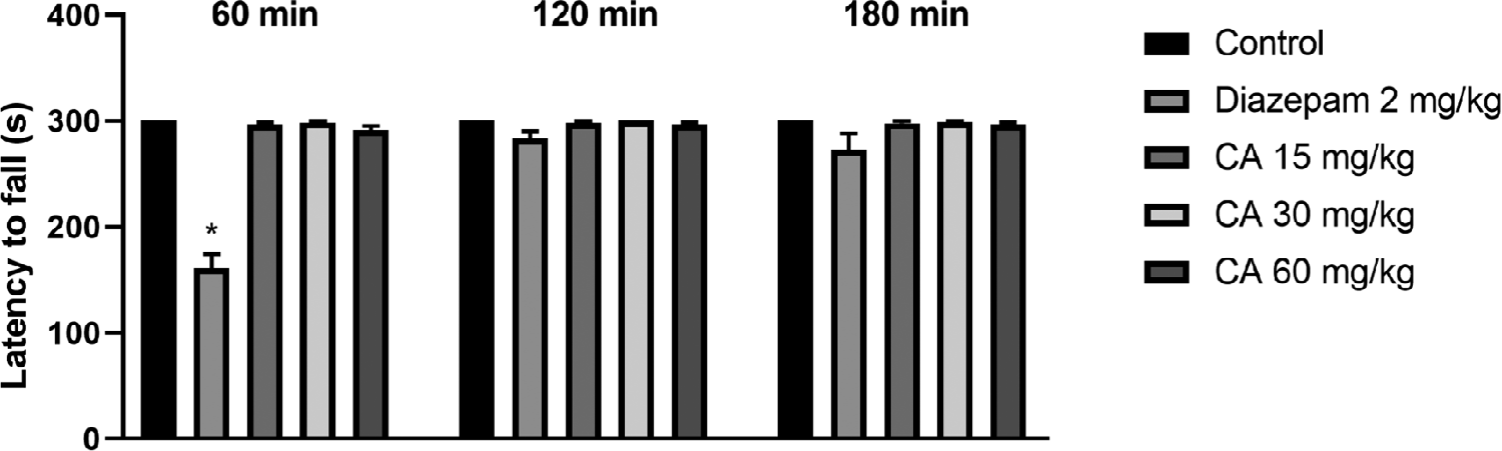
Effect of treatment with cinnamaldehyde (15, 30, and 60 mg/kg; p.o) and DZP (4 mg/kg; i.p) on the rota rod test at 60, 120, and 180 min. Legend: Each column represents mean ± standard deviation (*n* = 6), being compared by Kruskal–Wallis, followed by Dunn's post‐test. *p <0.01 versus control.

Treatment with cinnamaldehyde at doses of 15, 30, and 60 mg/kg had no effect on the time spent on the rotating bar (in seconds) at 30 min (296.7 ± 2.1; 298.5 ± 1.5; and 291.2 ± 4.0, respectively), 60 min (298.0 ± 2.0; 300.0 ± 0.0; and 296.7 ± 2.1, respectively), and 120 min (297.7 ± 2.3; 298.7 ± 1.3; and 296.8 ± 2.0, respectively) after treatment, when compared to the control group, which maintained a consistent residence time of 300.0 ± 0.0 s across all time points. These findings suggest the absence of myorelaxant activity for CA at the tested doses. As expected, diazepam (2 mg/kg) significantly reduced the time on the rotating bar at 30 min post‐administration (*p* = 0.0018). These results are shown in Figure 5.

### 2.5 Hot Plate Test

**FIGURE 6:**
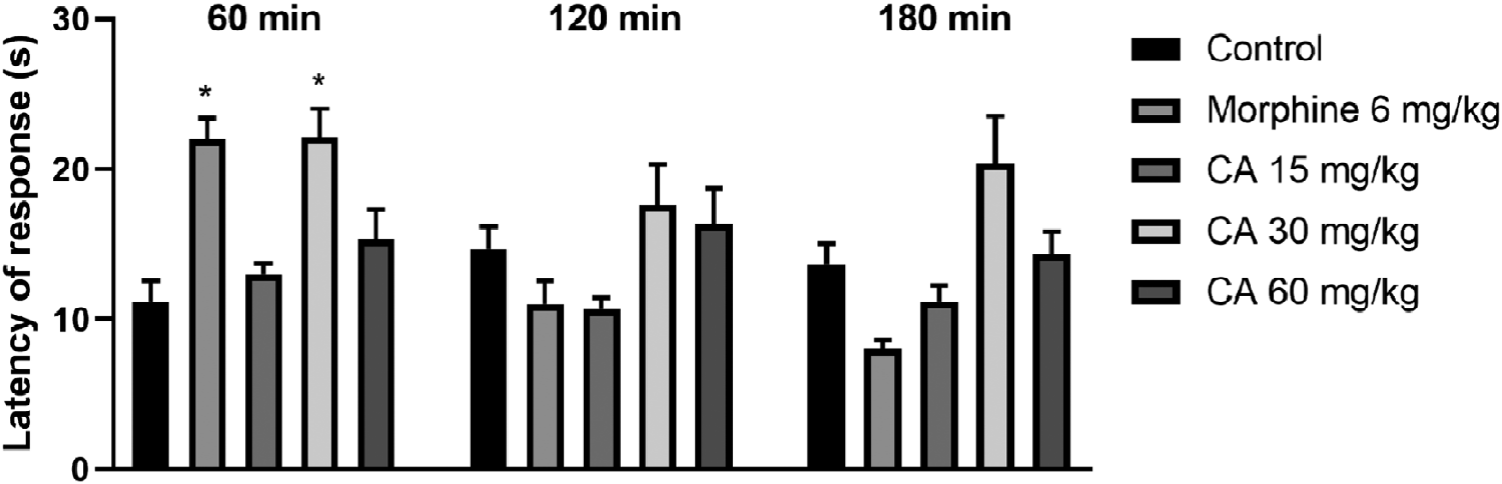
Effect of treatment with CA (15, 30, and 60 mg/kg; p.o) and morphine 6 mg/kg (i.p) on latency time in the hot plate test. Legend: Each column represents mean ± standard deviation (*n* = 6), being compared by analysis of variance (ANOVA), followed by Tukey's test. *p <0.01 versus control.

The results obtained in the hot plate test (Figure 6) demonstrate that CA promoted a significant increase in latency time (in seconds) only at the dose of 30 mg/kg, 60 min after administration, when compared to the control group (22.17 ± 1.9 versus 11.17 ± 1.4, *p* = 0.0003). This effect was similar to that observed with the standard drug morphine (22.0 ± 1.4). At other times, no statistical difference was observed in the increase in latency, suggesting a decrease in the antinociceptive effect as a function of time. At the other doses tested (15 and 60 mg/kg), CA did not demonstrate significant efficacy.

### 2.6 Formalin, Glutamate, and Capsaicin‐Induced Nociception

**FIGURE 7:**
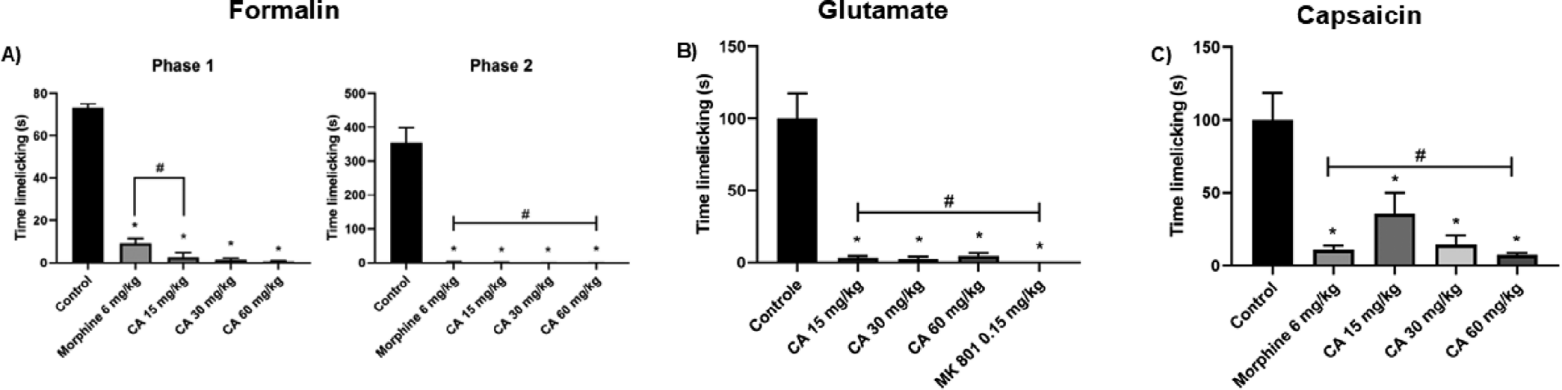
Effect of cinnamaldehyde treatment (15, 30, and 60 mg/kg; p.o.) on formalin (A), glutamate (B) and capsaicin‐induced (C) nociception test in mice. Legend: Each column represents the mean ± standard deviation (*n* = 6). Formalin: Phase 1 (ANOVA, *p* < 0.0001, *F* = 317.0); Phase 2 (ANOVA, *p* < 0.0001, *F* = 128.3); glutamate (ANOVA, *p* < 0.0001, *F* = 30.43); capsaicin (ANOVA, *p* < 0.0001, *F* = 12.54). Group comparisons were made using Tukey's test. *p < 0.01 versus control.

Cinnamaldehyde significantly reduced paw‐licking time (in seconds) during the first phase of the formalin test (neurogenic pain) at all tested doses: 15 mg/kg (2.66 ± 2.1, *p* < 0.0001), 30 mg/kg (1.50 ± 0.7, *p* < 0.0001), and 60 mg/kg (0.66 ± 0.3, *p* < 0.0001). The reference drug morphine (6 mg/kg) also produced a significant reduction in nociceptive behavior (9.00 ± 2.5, *p* < 0.0001). Compared to morphine, the 15 mg/kg dose of CA did not show a statistically significant difference, whereas the 30 mg/kg (*p* = 0.0424) and 60 mg/kg (*p* = 0.0199) doses demonstrated significantly greater antinociceptive effects. In the second phase of the test (inflammatory pain), CA reduced paw‐licking time by 99.90%, 99.85%, and 99.20% for the respective doses of 15 mg/kg (1.3 ± 1.1, *p* < 0.0001), 30 mg/kg (0.3 ± 0.2, *p* < 0.0001), and 60 mg/kg (0.5 ± 0.2, *p* < 0.0001), producing effects comparable to those of morphine (2.8 ± 1.1, *p* < 0.0001). These findings are described in Figure 7.

In the glutamate‐induced nociception test, CA administered at doses of 15, 30 and 60 mg/kg and MK‐801 at a dose of 0.15 mg/kg decreased paw licking time (in seconds) by 96.64 (3.3 ± 1.2, *p* < 0.0001), 97.64 (2.3 ± 1.8, *p* < 0.0001), 95.31 (4.6 ± 1.9, *p* < 0.0001) and 100% (0.0 ± 0.0, *p* < 0.0001), respectively, in relation to the control group, showing no statistical difference between them (Figure 7).

In the capsaicin‐induced nociception test, CA significantly reduced paw‐licking time (in seconds) at all tested doses. The reductions observed were 64.15% (54.67 ± 21.6, *p* = 0.0029) at 15 mg/kg, 85.68% (21.83 ± 9.8, *p* < 0.0001) at 30 mg/kg, and 92.67% (11.17 ± 1.8, *p* < 0.0001) at 60 mg/kg. No statistically significant differences were found between the CA‐treated groups or between any of these groups and the morphine‐treated group, suggesting a comparable efficacy across treatments. These findings are illustrated in Figure 7.

### 2.7 Opioid Receptor Involvement in Cinnamaldehyde Antinociceptive Effect

**FIGURE 8:**
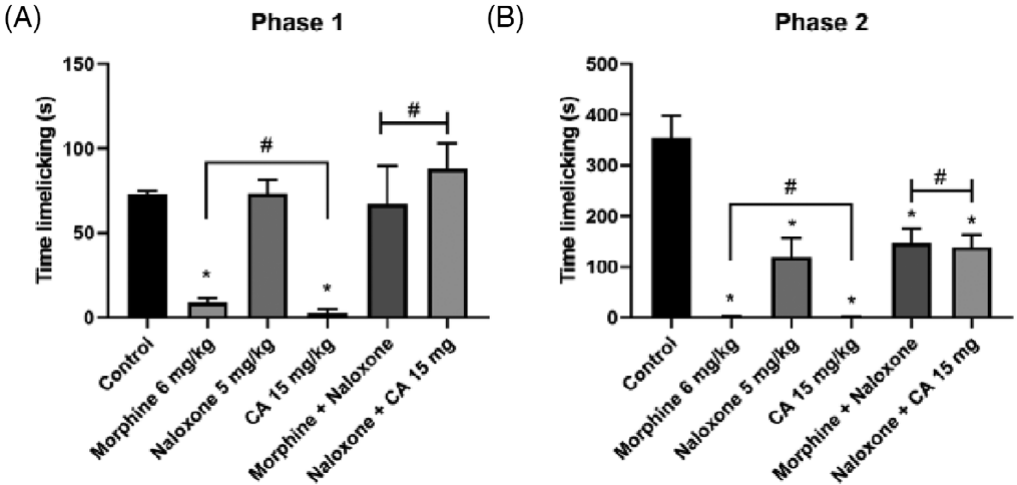
Effect of CA (15 mg/kg; p.o) and morphine (6 mg/kg; i.p) treatment with the opioid pathway antagonist (naloxone 5 mg/kg; s.c) in the two phases of the formalin test in mice (phase 1 a, phase 2 b). Legend: Each column represents mean ± standard deviation (*n* = 6), being compared by analysis of variance (ANOVA), followed by Tukey's test. *p<0.005 (a) and *p* <0.01 (b) vs naloxone.

To demonstrate the participation of the opioid system in the antinociceptive activity of CA, the formalin test was performed with the inclusion of the pathway antagonist, naloxone (Figure 8).

The prior administration of naloxone (5 mg/kg) promoted a reversal of the antinociceptive effect induced by CA (15 mg/kg) in the formalin‐induced neurogenic pain phase by 100%. The antinociceptive effect induced by CA was reversed in the presence of naloxone (*p* = 0.9425, naloxone vs. naloxone plus CA). As expected, naloxone was also able to reverse the antinociceptive effect promoted by morphine (6 mg/kg) from 88.91 (11.8 ± 3.56 s) to 8.49% (91.56 ± 31.88 s, *p* = 0.9991, naloxone vs. naloxone + morphine). In this assay, it is also possible to observe that in the second phase of the test, the use of naloxone, alone or associated with morphine or CA, did not promote complete reversal of the nociceptive effect.

Prior administration of naloxone (5 mg/kg) reversed (100%) the antinociceptive effect induced by CA (15 mg/kg) in the neurogenic phase of formalin‐induced pain (*p* = 0.9425; control vs. naloxone + CA). In the second phase (inflammatory phase) of the test, CA elicited an antinociceptive effect in the presence of naloxone (*p* < 0.0001; control vs. naloxone + CA). As expected, naloxone also reversed the antinociceptive effect of morphine (6 mg/kg), reducing the inhibition of nociceptive behavior from 88.91% (11.8 ± 3.56 s) to 8.49% (91.56 ± 31.88 s; *p* = 0.9991; control vs. naloxone + morphine). In this assay, it was also observed that, during the second phase of the test (inflammatory pain), naloxone, whether administered alone or in combination with CA or morphine, did not produce a complete reversal of the antinociceptive effect.

### 2.8 Adrenergic Receptor Involvement in Cinnamaldehyde Antinociceptive Effect

**FIGURE 9:**
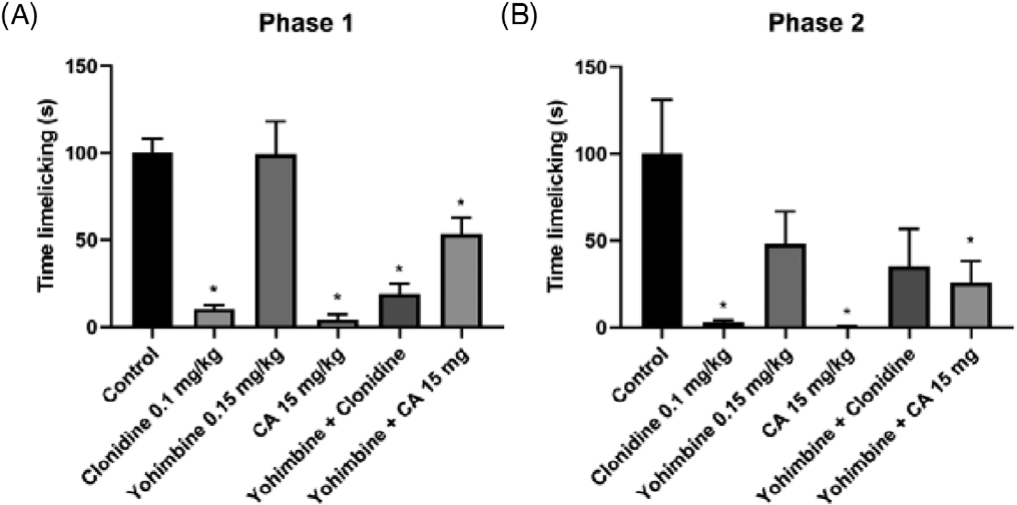
Effect of treatment of CA (15 mg/kg; p.o) and yohimbine (0.15 mg/kg; i.p) with the adrenergic pathway antagonist (clonidine hydrochloride 0.1 mg/kg; i.p) in the two phases of the formalin test in mice (phase 1 a, phase 2 b). Legend: Each column represents mean ± standard deviation (*n* = 6), being compared by analysis of variance (ANOVA), followed by Tukey's test. *p <0.01 versus control.

In the investigation of the adrenergic pathway through the formalin‐induced pain test, it was possible to observe a significant antinociceptive effect (paw licking time in seconds) of CA, a result similar to that of clonidine (*p* = 0.9997; clonidine vs. CA), an α2‐adrenergic agonist (Figure 9). When CA was associated with yohimbine, which is an antagonist of the adrenergic pathway, the antinociceptive effect was reduced from 95.97 (2.66 ± 2.1) to 46.73% (35.33 ± 6.5, *p* = 0.0212; control vs. CA + yohimbine) in the first phase of the test and from 99.62 (1.33 ± 1.1) to 66.13% (120.0 ± 39.5, *p* < 0.0001; control vs. CA + yohimbine) in the second phase.

After integrating the in silico and in vivo findings, the data suggest that the antinociceptive effect of CA may involve interactions with opioid receptors. Molecular docking indicates favorable binding and key interactions with κ‐ and δ‐opioid receptor sites, corroborating the possible participation of the opioid system in CA‐induced nociception modulation.

## 3 Discussion

The study confirms that CA exhibits antinociceptive effects in mice after oral administration, as behavioral screening demonstrated an increased latency in response to tail pressure, indicating reduced pain sensitivity [15]. This effect occurred at all tested doses without causing mortality, supporting the initial hypothesis. To further evaluate CA's effects on the nervous system, the open field test was used to assess emotional behavior in mice, particularly anxiety, sedation, or exploratory activity [16]. CA at 60 mg/kg significantly reduced ambulatory activity and rearing behavior, in a manner comparable to diazepam, indicating a decrease in exploratory behavior. Supporting this, CA has shown anxiolytic effects via the HPA axis and GABAergic modulation [17], with high doses possibly decreasing locomotor activity. Cinnamyl alcohol also showed anxiolytic behavior in mice [10]. In silico studies indicate cinnamic derivatives may interact with GABAA receptors, reinforcing this hypothesis.

To clarify whether the reduction in exploratory behavior observed in the open field was associated with motor impairment, the rotarod test was used to detect potential alterations in motor coordination. This method assesses motor coordination based on the mouse's ability to walk and balance on a rotating rod at a constant speed [19]. CA treatment did not alter the latency to fall from the rotating bar at any tested dose, indicating preservation of motor coordination and balance. In experimental models, substances with pronounced muscle relaxant or ataxic effects typically impair rotarod performance, which may confound the interpretation of nociceptive behaviors. Thus, the dissociation between reduced open field activity and preserved rotarod performance suggests that CA does not induce overt motor dysfunction or muscle relaxation. Rather, the observed behavioral profile supports the interpretation that CA modulates central motivational or emotional components without compromising motor coordination. Therefore, diminished open field activity at higher doses of cinnamaldehyde may reflect central behavioral modulation without indicating neuromuscular dysfunction [20].

The hot‐plate test revealed a nonlinear dose–response profile, in which only the intermediate dose of cinnamaldehyde (30 mg/kg) significantly increased nociceptive response latency, whereas both the lower (15 mg/kg) and higher (60 mg/kg) doses were ineffective. This inverted U‐shaped response suggests the existence of a dose‐dependent therapeutic window, a phenomenon frequently observed for centrally acting compounds and constituents of essential oils. At lower doses, cinnamaldehyde may fail to reach the pharmacodynamic threshold required to modulate central nociceptive pathways, while higher doses may recruit non‐specific central effects or compensatory mechanisms that counteract antinociception [21]. From a mechanistic perspective, this effect may be associated with the modulation of neural pathways involved in thermal pain processing, potentially through interactions with temperature‐sensitive ion channels of the TRP family, particularly TRPV1, which play a key role in thermal nociception [22]. In this context, molecular docking analyses revealed favorable binding energy values (−55.636) and interactions with critical TRPV1 residues (Arg557 and Ser512), which have been described as important for channel function, supporting a possible involvement of TRPV1 in the antinociceptive effects observed in the hot‐plate test [23].

However, to confirm the underlying mechanism, further investigations should be conducted using more specific experimental models of thermal hyperalgesia, such as the tail‐flick test and the Hargreaves model, which more precisely assess the thermal pain threshold and the efficacy of centrally acting analgesic agents [24].

To investigate the antinociceptive activity of CA, the formalin test was used, a model resembling clinical pain with two distinct phases [25]. The first phase involves neurogenic pain via C and Aδ fiber activation and mediator release. The second, inflammatory phase includes histamine, serotonin, prostaglandins, and bradykinin [26]. This test complements behavioral screening by confirming CA's effect in both pain phases.

CA significantly reduced paw‐licking time in both formalin test phases, indicating neurogenic and inflammatory pain relief, similar to morphine. Cinnamyl alcohol showed similar effects [10]. Anti‐inflammatory potential was also shown by cinnamon compounds [27] and by cinnamic acid in the test's second phase [28].

Glutamate is a major excitatory neurotransmitter released by both the central and peripheral nervous systems in response to nociceptive stimuli. Peripheral administration of glutamate directly activates primary afferent fibers, leading to the release of inflammatory mediators and neuropeptides, as well as activation of ionotropic and metabotropic glutamate receptors at peripheral, spinal, and supraspinal levels [29]. The nociceptive response induced by glutamate has been consistently associated with the activation of NMDA and non‐NMDA receptors in these regions [26].

In the present study, CA significantly reduced paw‐licking time in the glutamate‐induced nociception test, indicating an antinociceptive effect in this model. This behavioral finding was functionally comparable to the effect produced by MK‐801, a noncompetitive NMDA receptor antagonist, suggesting the involvement of glutamatergic signaling in CA‐induced antinociception. In silico analyses further supported this hypothesis by indicating a favorable molecular compatibility of CA with NMDA (−53.336) and AMPA (−63.071) receptor binding pockets, although these results should be interpreted as predictive rather than confirmatory.

Additional mechanisms may also contribute to this effect. CA has been reported to inhibit nitric oxide (NO) production through suppression of iNOS expression [30], which may attenuate glutamate‐driven excitation and RyR1‐mediated calcium release [31]. Similar antinociceptive and anti‐inflammatory profiles have been described for structurally related phenylpropanoids, such as 2‐allylphenol and ortho‐eugenol [32], supporting a shared pharmacological pattern within this class of compounds.

Continuing the investigation into the antinociceptive activity of various pungent agents, the capsaicin test was conducted. Capsaicin, the primary component found in red pepper and a well‐established agonist of the TRPV1 channel, is responsible for the characteristic burning sensation. It acts as a pro‐inflammatory agent by stimulating the release of neuropeptides such as neurokinin A, substance P, and calcitonin gene‐related peptide (CGRP) from primary afferent neurons, particularly within the trigeminal nerve.

TRPV1 channels are predominantly expressed in Aδ and C fibers, and their activation promotes Ca^2^^+^ influx, leading to membrane depolarization and neuronal excitation, which underlies the nociceptive response elicited by capsaicin [33]. For this reason, the capsaicin test is widely employed as a functional model to evaluate compounds that may interfere with TRPV1‐mediated nociceptive signaling.

In this context, CA significantly reduced nociceptive behavior in models sensitive to TRPV1‐mediated signaling, suggesting a possible contribution of this receptor to its antinociceptive profile. TRPV1 modulation is widely recognized as a relevant strategy for controlling hyperalgesia and neuropathic pain, due to its role in nociceptor excitability and in the release of pro‐inflammatory neuropeptides such as substance P and CGRP [34]. However, since no selective TRPV1 antagonists were employed, this interaction is discussed as a plausible, but not definitively established, mechanism.

Taken together, the use of multiple nociceptive models provides complementary evidence for the antinociceptive potential of CA and supports the involvement of interconnected pain‐modulating pathways [35].

After confirming CA's antinociceptive effect, its mechanism was investigated, focusing on the opioid system, a common target in pain research [36]. Opioid receptor activation closes presynaptic calcium channels, reducing neurotransmitter release, and opens potassium channels, causing postsynaptic hyperpolarization. This reduces the release of pain mediators like substance P [37], mimicking morphine's effects but potentially avoiding its side effects.

Naloxone reversed the antinociceptive effect of CA and morphine in the first test phase, suggesting the involvement of the opioid system. Naloxone is a well‐established non‐selective opioid receptor antagonist widely used in preclinical pain models to identify opioid system participation at a functional level, although it does not allow discrimination among specific receptor subtypes.

*In silico* modeling indicated that cinnamaldehyde shows predicted affinity for key opioid receptor sites, with a particular emphasis on κ (Ile294) and δ (Tyr308) receptors [38]. The observed hydrophobic interactions, typical of lipophilic compounds, are consistent with a binding pattern comparable to known opioid ligands, despite the simpler chemical structure of CA [13].

Additionally, compounds with structural similarity to cinnamaldehyde have been associated with analgesic and antinociceptive activity with a reduced incidence of adverse effects typically associated with strong μ‐opioid receptor activation, such as respiratory depression and dependence [39]. Within this pharmacological framework, cinnamaldehyde emerges as a pharmacologically relevant natural compound whose analgesic effects merit further mechanistic and translational investigation [40].

Furthermore, studies have highlighted the significant role of the noradrenergic system in descending inhibitory pain modulation. In the dorsal horn of the spinal cord, α_2_‐adrenergic receptors are found in primary nociceptive afferent fibers [41]. Activation of α_2_ receptors by descending noradrenergic pathways promotes an inhibitory regulatory effect on acute pain modulation, mediated by the Gi protein pathway, leading to inhibition of adenylate cyclase enzyme, consequently affecting cAMP formation and increasing cellular K^+^ efflux [42].

In the formalin test, CA showed significant antinociceptive effects similar to clonidine, an α_2_‐adrenergic agonist. Yohimbine, a selective α_2_ antagonist, reduced CA's effect from 95.97% to 46.73% (phase 1) and 99.62% to 66.13% (phase 2), indicating partial mediation via this pathway. As the effect wasn't fully reversed, other mechanisms likely contribute. Based on capsaicin test results, CA was further evaluated using the hot plate test, which measures latency to nocifensive behavior at 55°C [43].

Therefore, the results of this study suggest that CA possesses promising and effective antinociceptive activity against different types of nociceptive stimuli, both chemical and thermal. The data indicate that its mechanism of action is predominantly associated with modulation of the opioid pathway, although other systems may also contribute to the observed effect.

## 4 Experimental Section

### 4.1 Substance

Cinnamaldehyde (C_9_H_8_O), obtained from Merck KGaA—cinnamaldehyde, CAS No. 104‐55‐2), presents pale yellow in color, naturally found in the essential oil of cinnamon bark (*Cinnamomum zeylanicum* Blume), with a molecular weight of 132.16 g/mol, melting point −7°C, boiling point 246°C, density 1.049 at 20°C, partially soluble in water. The solution used in the study was prepared with 5% Tween and administered orally (p.o.).

### 4.2 Molecular Docking Analysis

Molecular docking simulations, within the Structure‐Based Drug Design (SBDD) approach, were carried out to contribute to the elucidation of the antinociceptive mechanism of action of cinnamaldehyde through its binding affinity to the targets involved in this effect. These targets included opioid receptors (Kappa (K) – 4DJH, Mu (μ) – 6DDF, and Delta (σ) – 6PT3); cyclooxygenase pathway (COX‐1 – 6Y3C, COX‐2 – 6COX); glutamatergic pathway (NMDA receptor – 4NF5, AMPA receptor – 5GZ0, and metabotropic glutamate receptor – 4OO9); adrenergic pathway (α2‐adrenergic receptor – 5FJV); and the TRPV channel – 5IS0. Docking simulations were performed using the 3D structures of the targets associated with these pathways, which are implicated in antinociceptive activity. The 3D structures of the proteins were retrieved from the Protein Data Bank (PDB) [44]. For comparison and evaluation of the compound's binding affinity to the studied targets, reference compounds were used in accordance with procedures applied in biological tests and guided by literature data. These compounds, along with descriptions of the proteins under study, are listed in Table S1.

The cinnamaldehyde molecule was drawn using Marvin Sketch v. 19.18 (https://chemaxon.com/marvin, accessed on July 24, 2024) and saved as a  .sdf file. Next, compound standardization was performed using ChemAxon's Standardizer software v. 21.2.0 (https://chemaxon.com/standardizer, accessed on July 24, 2024), where actions included the addition of hydrogen atoms, aromatic ring standardization, salt removal, and 3D structure conversion. This tool offers 40 predefined actions such as charge neutralization, fragment removal (e.g., salts), functional group recognition, and unified representation of tautomers and mesomers. There are two modes of use: an automatic one‐click standardization and a customizable mode where users can define the operations and their order [45]. After this step, the compounds were subjected to molecular docking.

The simulations were performed using Molegro Virtual Docker v.6.0.1 (MVD), with the predefined parameters. The coordinates of the molecular docking simulation site (Table S1) were determined using the co‐crystallized ligand, except for the cyclooxygenase 1 (COX‐1, 6Y3C) target, which was determined using the open‐access web platform Proteins Plus (Available at: https://proteins.plus/, accessed on July 24, 2024) [46], with the amino acid Tryptophan91, a critical residue of this target, as a reference [47]. The MoldDock SE (Simplex Evolution) algorithm was used with the following parameters: A total of 30 runs with a maximum of 2,000 iterations using a population of 50 individuals, 2,000 minimization steps for each flexible residual, and 2000 global minimization steps per run. A grid was set at 0.3 A, and the search sphere was set at a radius of 20 A. For ligand energy analysis, internal electrostatic interactions, internal hydrogen bonds, and sp2–sp2 torsions were evaluated. Prior to molecular docking simulations, a redocking procedure was performed, in which the root mean square deviation (RMSD), calculated from the obtained poses, was evaluated to verify the reliability of the docking protocol. The RMSD value indicates the degree of proximity between the predicted conformation and the experimental structure, with redocking considered successful when the RMSD is less than 2.0 Å. The selected pose was defined as the conformation exhibiting the lowest binding energy among the generated docking poses. Molecular interaction visualization and image generation were carried out using Discovery Studio Visualizer v20.1.0.19295 ‐ https://discover.3ds.com/discovery‐studio‐visualizer‐download.

### 4.3 Animals

Male Swiss mice (25–35 g), 2–3 months old, from the Prof. Dr. Thomas George vivarium (IPEFarM/UFPB) were randomly separated into appropriate cages with free access to a standard pellet diet (Quimtia, Brazil) and water. The animals were kept at a monitored temperature (21 ± 1°C), under a 12‐h light/dark cycle. All studies were carried out between 12:00 a.m. and 6:00 p.m., with the experiments previously approved by the Ethics Committee on the Use of Animals (CEUA #8170290422) of the Federal University of Paraíba, Brazil. All reagents were purchased from Sigma‐Aldrich (St. Louis, MO, USA). The drugs were administered at a dosage of 0.1 mL/10 g of body weight.

Independent groups of animals were used for each behavioral test to prevent potential bias or physiological interference between assays. The animals used in the behavioral screening, open field, rotarod, and nociception tests (formalin, glutamate, capsaicin, and hot plate) were not reused across experiments. After the completion of each test, the animals were humanely euthanized in accordance with ethical guidelines approved by the institutional animal care and use committee.

Sample size determination was based on statistical criteria using G Power software (version 3.1). A two‐tailed *t*‐test was considered, with an effect size of 1.8, a significance level of 5% (*α* = 0.05), and a statistical power of 80% (1 – β = 0.80). Based on these parameters, the analysis indicated that a minimum of six animals per group was required to detect significant differences with adequate power. Specifically for the behavioral screening test, three animals per group were used, following the recommendation of the protocol described by Almeida et al. [15].

### 4.4 Behavioral Screening

Initially, a behavioral screening was conducted to assess stimulant and depressant effects on the central and autonomic nervous systems, to evaluate the effect of CA four hours after oral administration. Animals in the control group (*n* = 3) received the vehicle, while the test groups received cinnamaldehyde at three doses (*n* = 3 for each dose). These doses were determined based on previous studies, which reported an LD_50_ of 2225 mg/kg [48]. Behavioral parameters were evaluated according to the protocol proposed by Almeida et al. [15].

### 4.5 Open Field Test

The animals were submitted to the open field test and evaluation for 5 min. For the experimental procedure, groups of six mice were used. CA (15, 30 e 60 mg/kg; p.o.); vehicle (saline solution; p.o.), and diazepam (DZP, 2 mg/kg; i.p.) were administered 60 min before the start of the test. After treatment, each animal was individually submitted to the device. The total number of quadrants covered (crossings), head rearing, and time spent on grooming were recorded [49].

### 4.6 Locomotor Activity‐Rotarod Test

The rotarod test checks motor coordination and muscle relaxation produced by central nervous system depressant drugs in animals [50]. The animals were preselected 24 h before the experiments, and those that were unable to remain on the rotating bar (2.5 cm in diameter, with a frequency of 7 rpm) for 1 min were excluded. Three groups were formed (n = 6): CA (15, 30, and 60 mg/kg; p.o.); vehicle (saline solution; p.o.), and DZP (4 mg/kg; i.p.). The animals were individually submitted to the rotarod test at 60, 120, and 180 min after treatments, and the time (s) they remained on the bar.

### 4.7 Hot Plate Test

The animals were pre‐selected, and those considered suitable presented a pain response time of less than 10 s (s) when placed on the hot plate apparatus (Insight, Ribeirão Preto, SP, Brazil) at 55 ± 1°C [51]. The selected mice were pre‐treated with vehicle (saline solution; p.o.), CA (15, 30, and 60 mg/kg; p.o.), or morphine (6 mg/kg; i.p.), and they were individually placed on the hot plate at 0.5, 1, and 2 h after initial treatment. The registered parameter was the latency time to jump or lick the hind paws. To minimize animal paw tissue destruction, the time on the plate did not exceed 30 s.

### 4.8 Formalin, Glutamate, and Capsaicin‐Induced Nociception

The nociception protocols were performed on the mice by s.c. injection of 20 µL 2% formalin, 20 µl of glutamate (30 mM), or capsaicin (20 µL, 2.5 µg) in the subplantar region of the right hind paw. Mice (*n* = 6, per group) were treated with either vehicle (saline solution; p.o.), CA (15, 30 e 60 mg/kg; p.o.), or morphine (6 mg/kg; i.p.), or MK‐801(0.15 mg/kg, i.p.) at 1 h or 30 min before specific algogen administrations. Formalin test: The neurogenic phase occurs within 0–5 min after the administration of formalin and is followed by a latency period of about 10 min. An inflammatory phase occurs within 15–30 min after formalin administration [52]. Glutamate test: Mice were observed individually for 15 min following glutamate injection [25]. Capsaicin test: Animals were assessed 20 min after injection of capsaicin [53]. The animals were observed individually in mirrored chambers (25 × 25 × 25 cm) to allow an unobstructed view of the paw region. For the three tests, the nociceptive behavior was assessed during the time spent licking their injected paws.

### 4.9 Opioid Receptor Involvement in Cinnamaldehyde Antinociceptive Effect

To identify possible opioid receptor involvement in the antinociceptive effect of CA, six groups were used: the vehicle (saline solution; p.o), CA (15 mg/kg; p.o.), morphine (6 mg/kg; i.p.), and the remaining groups received the same prior treatments. However, 15 min earlier, they were given naloxone (5 mg/kg; s.c.) an opioid receptor antagonist. The formalin test was then performed.

### 4.10 Adrenergic Receptor Involvement in Cinnamaldehyde Antinociceptive Effect

To identify possible α2‐adrenergic receptor involvement in the antinociceptive effect of CA, six groups were used: the vehicle (saline solution; p.o), CA (15 mg/kg; p.o.), clonidine hydrochloride (0.1 mg/kg; i.p.), and the remaining groups received the same prior treatments. However, 15 min earlier, they were given yohimbine (0.15 mg/kg; i.p.), an adrenergic receptor antagonist. The formalin test was then performed.

### 4.11 Statistical Analysis

The results were analyzed using one‐way Analysis of Variance (ANOVA), followed by the Tukey test (for parametric measurements), and Kruskal‐Wallis, followed by Dunn's test (for non‐parametric measurements). The Shapiro‐Wilk test was used to evaluate data distribution. The values obtained were expressed as means ± standard deviation (S.D.). A level significance of 5% was adopted. The data collected was analyzed using GraphPad Prism 8.0.1.

## 5 Conclusion

The present study demonstrated that cinnamaldehyde exerts significant antinociceptive effects in acute nociception models. The findings suggest a possible association with the modulation of central pathways involving opioid and adrenergic systems, based on behavioral assays, with molecular docking analyses providing supportive *in silico* evidence. The absence of motor impairment at the tested doses indicates a favorable acute safety profile under the experimental conditions employed.

Thus, cinnamaldehyde stands out as a promising molecule in acute pain therapy, justifying the continuation of preclinical studies aimed at deepening its mechanisms of action, toxicological profile, and efficacy in chronic pain models.

## Author Contributions

This study was designed by R.R., R.C., and M.S. The experiments were performed by R.R., H.P., A.D., M.R., H.A., P.S., N.S., L.S., and M.S. The data were analyzed by R.R, and the results were critically examined by all authors. All authors have approved the final version of the manuscript and agree to be accountable for all aspects of the work.

## Funding

This research was supported by the Fundação de Apoio à Pesquisa do Estado da Paraíba (FAPESQ).

## Ethics Statement

The protocols in this research were previously approved by the Ethics Committee on the Use of Animals (CEUA #8170290422) of the Federal University of Paraíba, Brazil.

## Conflicts of Interest

The authors declare no conflicts of interest.

## Supporting information




**Supporting File**: cbdv71255‐sup‐0001‐SuppMat.docx.

## Data Availability

The data that support the findings of this study are available from the corresponding author upon reasonable request.
