# Supplementary material for: Antinociceptive Effect of Cinnamaldehyde in Male Mice: Investigation of the Mechanisms of Action Through In Silico and In Vivo Approaches
Source: Chem Biodivers. 2026 Apr 27;23:e03228. doi: 10.1002/cbdv.202503228 (PMC13112334; doi:10.1002/cbdv.202503228)
Supplement: Supplementary file 1 — Supporting File: cbdv71255‐sup‐0001‐SuppMat.docx. [file CBDV-23-e03228-s001.docx]

**Supplementary material to:**

**Antinociceptive Effect of Cinnamaldehyde in Male Mice: Investigation of the Mechanisms of Action through *in silico* and *in vivo* approaches**

Renaly I. de A. Rêgo^[a]^, Hugo F. O. Pires^[a]^, Arthur L. Dias^[a]^, Maria Caroline R. B. Remigio^[a]^, Humberto H. N. de Andrade^[b]^, Pablo R. da Silva^[a]^, Natália F. de Sousa^[a]^, Luciana Scotti^[a]^, Marcus T. Scotti^[a]^, Mirian G. S. S. Salvadori^[a]^, Ricardo D. de Castro^[a,*]^

[a] Department: Postgraduate Program in Natural and Synthetic Bioactive Products, Center of Health Science.

Institution: Federal University of Paraíba (UFPB).

Adress 1. Jardim Universitário, S/N – Campus I – Castelo Branco, João Pessoa-PB, 58051-900, Brazil.
E-mail: [renaly.ivyna@hotmail.com](mailto:renaly.ivyna@hotmail.com), [hugofernandes763@gmail.com](mailto:hugofernandes763@gmail.com), [arthurlinsd@gmail.com](mailto:arthurlinsd@gmail.com), [carolinebezerra.mcrb@gmail.com](mailto:carolinebezerra.mcrb@gmail.com), [pablorayff@ltf.ufpb.br](mailto:pablorayff@ltf.ufpb.br), [nataliafsousa@ltf.ufpb.br](mailto:nataliafsousa@ltf.ufpb.br), [luciana.scotti@gmail.com](mailto:luciana.scotti@gmail.com). [mtscotti@ccae.ufpb.br](mailto:mtscotti@ccae.ufpb.br), [mirian.salvadori@gmail.com](about:blank), [rcastro@ccs.ufpb.br](mailto:rcastro@ccs.ufpb.br)

[b] Department: Institute of Research in Drugs and Medicines.

Institution: Federal University of Paraíba (UFPB).

Adress 2. Jardim Universitário, S/N – Campus I – Castelo Branco, João Pessoa-PB, 58051-900, Brazil.
E-mail: [humbertohugo_92@hotmail.com](mailto:humbertohugo_92@hotmail.com)

**Corresponding author**: Ricardo Dias de Castro, Department of Postgraduate Program in Natural and Synthetic Bioactive Products, Center of Health Science, Federal University of Paraíba (UFPB), Jardim Universitário, S/N – Campus I – Castelo Branco, João Pessoa-PB, 58051-900, Brazil. Tel: +558332167502; E-mail: [rcastro@ccs.ufpb.br](mailto:rcastro@ccs.ufpb.br)

**Supplementary Material**

**Table 01:** Control compounds used in Molecular Docking simulations

| **Pathway** | **Receptor** | **PDB** | **Control** | **Co-crystallized ligand** | **Coordinates** |
| --- | --- | --- | --- | --- | --- |
| Opioid | K | 4DJH | Morphine | Carboxamide derivative  (JDC) | X: 3.68  Y: -24.01  Z: 59.47 |
| Opioid | µ | 6DDF | Morphine | Peptide  (PRD_002308) | X: 119.90  Y: 152.85  Z: 144.19 |
| Opioid | σ | 6PT3 | Deltorphin II | Ethylbenzamide derivative  (OWY) | X: 4.30  Y: -40.90  Z: -46.54 |
| COX | COX-1 | 6Y3C | Ibuprofen Dexamethasone | -  Coordinates based on the amino acid Trp91 | X: -34.02  Y: -20.24  Z: 13.21 |
| COX | COX-2 | 6COX | Indomethacin | Pyrazole derivative  (S58) | X: 23.58  Y: 23.34  Z: 47.83 |
| Glutamatergic | NMDA | 4NF5 | Disocilpine | Norvaline derivative  (2JJ) | X: 20.93  Y: -18.17  Z: 50.60 |
| Glutamatergic | AMPA | 5ZG0 | Disocilpine | Thiadiazine derivative  (9C3) | X: 23.58  Y: 56.17  Z: 19.99 |
| Glutamatergic | Metabotropic Glutamate | 4OO9 | MGS-0039 | Mavoglurant  (2U8) | X: -22.75  Y: -5.19  Z: 42.96 |
| Adrenergic | α2 adrenergic | 5FJV | Clonidine | Epibatidine  (EPJ) | X: 12.73  Y: 4.10  Z: 0.49 |
| Ion channel | TRPV1 | 5IS0 | Eriodictyol | Capsazepine  (6ET) | X: 138.18  Y: 108.14  Z: 102.58 |

**Table 02:** Affinity score values of the compound Cinnamic Aldehyde on the targets under study

| **Pathway** | **Receptor** | **PDB ID** | **Cinnamic Aldehyde** | **Positive control** | **PDB ligand** | **RMSD** |
| --- | --- | --- | --- | --- | --- | --- |
| Opioid | K | 4DJH | -61.215 | -100.819 | **-148.922** | 0.24 |
| Opioid | µ | 6DDF | -40.097 | -86.171 | **-124.081** | 0.29 |
| Opioid | σ | 6PT3 | -68.265 | **-204.289** | -141.39 | 0.28 |
| COX | COX-1 | 6Y3C | **-60.324*** | **-84.678** | **-76.700*** | - |
| COX | COX-2 | 6COX | -54.476 | -127.393 | **-144.23** | 0.30 |
| Glutamatergic | NMDA | 4NF5 | **-53.336*** | **-62.207*** | **-111.616** | 0.23 |
| Glutamatergic | AMPA | 5ZG0 | **-63.071*** | **-77.483*** | **-137.9** | 0.16 |
| Glutamatergic | Metabotropic Glutamate | 4OO9 | -72.734 | -132.367 | **-164.588** | 0.18 |
| Adrenergic | α2 adrenergic | 5FJV | **-44.978*** | **-46.999*** | **-62.981** | 0.19 |
| Ion channel | TRPV1 | 5IS0 | **-55.636*** | **-87.283** | **-74.354*** | 1.38 |

**Legend:** In bold are the compounds that presented the lowest energy, and in bold and with an asterisk are the targets in which the test compound presented an affinity score lower than or close to at least one of the controls under study.

**Table 03:** Layer score values of the cinnamic aldehyde compound in the targets under study after performing DSC normalization

| **Pathway** | **Receptor** | **PDB ID** | **Cinnamic Aldehyde (Score)** | **Cinnamic Aldehyde (DSC)** | **Positive control (Score)** | **Positive control (DSC)** | **PDB ligand (Score)** | **PDB ligand (DSC)** |
| --- | --- | --- | --- | --- | --- | --- | --- | --- |
| Opioid | K | 4DJH | -61.215 | **-0.463** | -100.819 | -0.353 | -148.922 | -0.319 |
| Opioid | µ | 6DDF | -40.097 | **-0.303** | -86.171 | -0.301 | -124.081 | -0.241 |
| Opioid | σ | 6PT3 | -68.265 | **-0.516** | -204.289 | -0.260 | -141.39 | -0.291 |
| COX | COX-1 | 6Y3C | -60.324 | **-0.456** | -84.678 | -0.41 | -76.700 | -0.195 |
| COX | COX-2 | 6COX | -54.476 | **-0.412** | -127.393 | -0.356 | -144.23 | -0.323 |
| Glutamatergic | NMDA | 4NF5 | -53.336 | -0.403 | -62.207 | -0.281 | -111.616 | **-0.566** |
| Glutamatergic | AMPA | 5ZG0 | -63.071 | **-0.477** | -77.483 | -0.350 | -137.9 | -0.433 |
| Glutamatergic | Metabotropic Glutamate | 4OO9 | -72.734 | **-0.550** | -132.367 | -0.349 | -164.588 | -0.525 |
| Adrenergic | α2 adrenergic | 5FJV | **-44.978** | -0.340 | -46.999 | -0.204 | -62.981 | -0.301 |
| Ion channel | TRPV1 | 5IS0 | -55.636 | **-0.420** | -87.283 | -0.302 | -74.354 | -0.197 |

**Legend:** In bold, the compound with the lowest binding energy.

**Molecular weight:** cinnamic aldehyde (132.16g/mol), morphine (285.34g/mol), JDC (465.62g/mol), PRD_002308 (513.593g/mol), deltorphin II (782.9g/mol), OWY (485.65g/mol), ibuprofen (206.28g/mol), dexamethasone (392.5g/mol), indomethacin (357.8g/mol), S-58 (446.24g/mol), disocilpine (221.30g/mol), 2JJ (197,13g/mol), 9C3 (318,39g/mol), MGS-0039 (378.2g/mol), 2U8 (313,38g/mol), clonidine (230.09g/mol), EPJ (208,684g/mol), muscimol (114.10g/mol), V8D (244,29g/mol), baclofen (213.66g/mol), 2BV (408,28g/mol), quimpirole (219.33g/mol), G6O (249.30g/mol), GMJ (375,85g/mol), PD168077 (334.4g/mol), AQD (388,90g/mol), eriodictyol (288.25g/mol), 6ET (376,90g/mol).

**Table 04:** Behavioral changes observed in animals treated with CA at different doses (15, 30 and 60 mg/kg). [(-) decreased effect, (+) present effect, (++) intense effect] n=4

| **Dose (mg/kg; p.o)** | **Observation time (min)** | **Behavioral effects** | **Analgesia** |
| --- | --- | --- | --- |
| **CA 15** | **30** | **-** | |
|  | **60** | **+** | |
|  | **120** | **-** | |
|  | **180** | **-** | |
|  | **240** | **-** | |
| **CA 30** | **30** | **-** | |
|  | **60** | **+** | |
|  | **120** | **+** | |
|  | **180** | **-** | |
|  | **240** | **-** | |
| **CA 60** | **30** | **+** | |
|  | **60** | **+** | |
|  | **120** | **+** | |
|  | **180** | **+** | |
|  | **240** | **-** | |

**Legend:** (-) absence of effect (+) presence of effect
